# Retinoids in Embryonic Development

**DOI:** 10.3390/biom10091278

**Published:** 2020-09-04

**Authors:** Michael Schubert, Yann Gibert

**Affiliations:** 1 CNRS, Laboratoire de Biologie du Développement de Villefranche-sur-Mer, Institut de la Mer de Villefranche, Sorbonne Université, 181 Chemin du Lazaret, 06230 Villefranche-sur-Mer, France; 2 Department of Cell and Molecular Biology, University of Mississippi Medical Center, Jackson, MS 39216, USA; ygibert@umc.edu

**Keywords:** animal models, developmental mechanisms, epigenetic regulation, genomic and non-genomic processes, origin and evolution of retinoic acid functions, receptor–ligand interactions, retinoid metabolism, retinoid pharmacology, retinoid receptors, vitamin A-dependent signaling

Retinoids constitute a class of compounds chemically related to vitamin A [1] that play major roles as signaling molecules during vertebrate embryogenesis [2,3,4] and invertebrate development [5,6,7]. The signaling functions of endogenous retinoids are mediated mainly by the acidic metabolite of vitamin A, retinoic acid (RA), and heterodimers of two nuclear receptors: the retinoic acid receptor (RAR) and the retinoid X receptor (RXR) [8]. RAR and RXR heterodimerize on DNA regulatory sites, so-called retinoic acid response elements (RAREs) [9], and, in the presence of RA, mediate the activation of target gene expression [10]. Recent studies have revealed a number of novel roles for RA-dependent signaling during development, some of which are controlled by alternative molecular mechanisms, such as the direct repression of a target gene, *Fgf8*, by liganded RAR/RXR heterodimers [11].

The scope of this special issue is to provide additional insights into the diversity of retinoid signaling during both vertebrate and invertebrate development. The collection includes six original research papers and six review articles from well-known experts in the field and provides the interested reader with specific examples of retinoid functions in different biological systems. Importantly, the articles both highlight the most recent results and illustrate perspectives for future research. This special issue thus furthers our understanding of retinoid biology, accumulated in the course of the last 30 years, and presents challenges that will need to be addressed by the scientific community in the near future.

The first research article by Bellutti et al. reassesses the roles of endogenous RA and of CYP26B1-dependent RA degradation in mouse fetal gonads. The authors show that some effects of RA are not recapitulated by invalidation or stimulation of CYP26B1 activity. Altogether, their results suggest that CYP26B1 does not exclusively function as an RA-metabolizing enzyme in mouse fetal gonads and that RA merely amplifies the expression of *Stra8*, the key regulator of meiotic entry, rather than inducing it [12]. Sarang et al. use knock-out approaches in mice to assess the role of retinol saturase (*RetSat*) in the phagocytic removal of apoptotic cells. They find that the genetic loss of *RetSat* leads to impaired clearance of apoptotic cells and the development of mild autoimmunity. Their results further indicate that the requirement of RetSat for the differentiation of monocytes and macrophages is independent of its enzymatic capacity to produce dihydroretinol [13].

The special issue also features several research articles characterizing the functions of RA signaling in non-canonical models. Viera-Vera and García-Arrarás, for example, describe the roles of RA during intestinal regeneration of the sea cucumber *Holothuria glaberrima*. This study reports that the pharmacological inhibition of RA signaling during intestinal regeneration results in a significantly smaller intestinal rudiment and reduces both cell division and cell dedifferentiation. Taken together, their work identifies similarities between the roles played by RA signaling during regeneration in echinoderms and other animal systems [14]. The research reported by Yamakawa et al. also focuses on RA-dependent processes in echinoderms. More specifically, this article investigates RA signaling functions during metamorphosis of the feather star *Antedon serrata*. Based on pharmacological treatments, the work by Yamakawa et al. demonstrates that RA signaling is required for the regulation of metamorphosis in feather star larvae. When interpreted in a comparative context, these results indicate that RA signaling already functioned as a regulator of metamorphosis in the last common ancestor of all echinoderms [15].

Johnson et al. use individual motoneurons of the gastropod *Lymnaea stagnalis* to study the effects of RA on growth cone guidance. They show that inhibition of two Rho GTPases, Cdc42 and Rac, prevent growth cone turning toward RA and induce a shift in growth cone responsiveness to RA from attraction to repulsion. They further demonstrate that these effects are dependent on the RA isomer (all-*trans* or 9-*cis* RA) used to induce the turning. Taken together, these results strongly suggests that Cdc42 and Rac act as downstream effectors in the signal transduction pathway controlling the directional mobility of growth cones in response to RA [16]. Contrasting the other research articles that are focused on a specific model organism, Fonseca et al. explore the phylogenetic distribution of RXRs in the animal tree of life. They further survey the capacity of RXRs from different animals to bind the RA isomer 9-*cis* RA and the xenobiotic compound tributyltin (TBT) and find that 9-*cis* RA and TBT can activate RXRs from more animal phyla than initially anticipated [17].

The collection of review articles of the special issue features a comprehensive summary by Belyaeva et al. of our current understanding of the enzymes producing all-*trans*-retinaldehyde for the biosynthesis of RA. These enzymes, called retinol dehydrogenases, regulate the flux from retinol to retinaldehyde and are thus critical for maintaining RA homeostasis in vivo. The article focuses on physiologically relevant retinol dehydrogenases and discusses enzymatic properties, tissue distribution as well as possible interactions with other components of the retinoid metabolism and signaling network [18]. The review by Endo et al. addresses the roles of RA in germ cell development of the mammalian ovary and testis. The article discusses both germ cell development in fetal gonads and the development of germ cells after birth [19].

Mezquita and Mezquita review the involvement of RA in cell proliferation and differentiation. The article provides a summary of the context-dependent functions of RA in regulating either the proliferation of stem and progenitor cells or the induction of cell differentiation. The authors argue that an understanding of the signaling pathways controlled by RA is crucial to improve the therapeutic potential of retinoids in regenerative medicine and cancer [20]. Draut et al. also discuss the roles of RA in cell fate choices, but focus on the development of cranial, axial and caudal structures as well as on regenerative processes. Albeit covering results from various vertebrates, the main focus of this article is on zebrafish (*Danio rerio*), hence highlighting the utility of this model for studying RA signaling [21].

The review by Feneck and Logan provides a summary of the earliest events of limb development, paying particular attention to the involvement of RA. The article highlights some of the challenges involved in studying the developmental roles of RA. These include the analysis of processes controlled by a molecule that diffuses freely across the embryo and the creation of an embryonic environment completely devoid of RA. The authors propose a combinatorial approach of different experimental strategies to dissect the various roles played by RA during embryonic development [22]. Fernandes-Silva et al. review the involvement of RA signaling in lung development, covering the different phases of lung ontogeny as well as the formation of the pulmonary vascular system. The article further discusses interactions of RA signaling with other developmental signaling pathways during organogenesis of the lung [23].

Taken together, this compilation illustrates the diverse roles played by RA signaling during embryogenesis in both vertebrates and invertebrates. The articles also highlight the importance of a tight regulation of endogenous RA signaling levels during development, with deregulations causing a wide variety of malformations, such as, to name but a few, skeletal defects, impaired vision, metabolic disorders, limb malformations and respiratory diseases. Yet, when browsing through the articles, it also becomes apparent that there are still numerous unresolved questions about RA and its biological functions. We thus hope that this special issue will encourage additional, deeper analyses of the roles played by RA and other retinoids during development and beyond.

We further argue that retinoid signaling should not only be studied using the latest technologies, but also in a wider variety of animal systems. As much as there is power in the newest techniques and methodologies, there is potential in extending the scope to as many different model systems as possible. In sum, although scientists have been studying retinoids for decades, we might still just be starting to grasp the immense diversity of the biological functions of these molecules. We hope that this special issue can help inspire the younger generation of scientists to embark with us on the journey to explore the functions of retinoids at the genetic, physiological, developmental, cellular, molecular, genomic and evolutionary levels, in as many different animal systems as possible.
